# Metabolizable energy and amino acid digestibility in spray-dried animal plasma using broiler chick and precision-fed rooster assays

**DOI:** 10.1016/j.psj.2022.101807

**Published:** 2022-02-25

**Authors:** H.V.N. Khadour, B.W. Parsons, P.L. Utterback, J.M. Campbell, C.M. Parsons, J.L. Emmert

**Affiliations:** ⁎ Department of Animal Sciences, University of Illinois at Urbana-Champaign, IL 61801, USA; # APC LLC, Ankeny, IA 50021, USA

**Keywords:** amino acid digestibility, metabolizable energy, poultry, spray-dried animal plasma

## Abstract

Four experiments were conducted to determine ME and amino acid (**AA**) digestibility of spray-dried animal plasma (**SDAP**) and soybean meal (**SBM**). The 48-h precision-fed adult rooster assay was used in 2 experiments; TME_n_ and standardized AA digestibility were determined using conventional and cecectomized roosters, respectively, 50 weeks of age and weighing approximately 2,200 g. Eight individually-caged roosters (4 per diet) were fasted for 26 h, then precision-fed 30 g of SDAP mixture (containing 50% corn) or SBM mixture (containing 50% corn). The TME_n_ and AA digestibility for SDAP and SBM were calculated by the difference procedure. The TME_n_ for SDAP was greater (*P* < 0.05) than SBM (3,743 and 2,669 kcal/kg DM, respectively). Similarly, mean AA digestibility of SDAP was greater (*P* < 0.05) than SBM (94 and 86%, respectively). Two assays were conducted using Ross male broilers to determine AME_n_ and apparent (**AIAAD**) and standardized (**SIAAD**) ileal AA digestibility of SDAP and SBM. A 3 × 2 factorial arrangement of treatments was used to determine AME_n_; 126 chicks (6 replicate pens of 7 chicks) were fed a corn-SBM-based reference diet, a diet containing 30% SDAP, or a diet containing 30% SBM from d 7 to 10 and 18 to 21. A 2 × 2 factorial arrangement of treatments was used to determine AIAAD and SIAAD; 168 chicks (12 replicate pens of 7 chicks) were fed a semi-purified diet containing 25% SDAP or a semi-purified, isonitrogenous diet containing 41% SBM from d 7 to 10 and 18 to 21. The AME_n_ for SDAP was greater (*P* < 0.05) than SBM at d 10 (3,851 and 2,089 kcal/kg DM, respectively) and d 21 (4,239 and 2,849 kcal/kg DM, respectively). The second assay showed an increase (*P* < 0.05) in AIAAD and SIAAD for SDAP compared with SBM at d 10 (mean SIAAD for SDAP and SBM were 96% and 84%, respectively) and d 21 (97% and 87%, respectively). Regardless of assay or age, these results indicate SDAP is a highly digestible feed ingredient with high ME and AA digestibility.

## INTRODUCTION

There is ongoing interest in the inclusion of feed ingredients containing highly digestible protein contents in poultry diets due to their ability to supply substantial amounts of essential amino acids (**AA**), reduce nitrogen excretion, and improve gastrointestinal health (Akhter et al., 2008). Spray-dried animal plasma (**SDAP**) is commonly derived from bovine and porcine origins and is a highly digestible protein source with a desirable AA profile (Castelló et al., 2004; Torrallardona, 2009).

Cost has typically limited the use of SDAP in poultry diets, but Henn et al. (2013) found that SDAP could improve broiler performance, particularly during the starter phase when birds were raised under challenging conditions caused by the reuse of litter from a previous flock with coccidiosis. Beski et al. (2015). fed SDAP at dietary levels up to 2% to broilers during the starter phase and noted improved feed efficiency that persisted through the grower and finisher phases, when SDAP was no longer being fed. Similarly, Beski et al. (2016) found beneficial effects, including improved BW and feed conversion ratio, associated with feeding dietary inclusion levels of 1 or 2% during the first 10 d posthatch.

Benefits of feeding SDAP have also been noted in broiler trials conducted under challenging conditions, typically associated with used litter or disease challenges. Bregendahl et al. (2005) reported benefits of feeding 2% SDAP from 1 to 42 d of age to broilers raised on soiled litter; effects included improved growth rate, feed conversion, breast-meat yield, and flock uniformity. In a study conducted with broilers with necrotic enteritis, feeding SDAP at levels of 1, 0.5, and 0.25% during the starter, grower, and finisher phases, respectively, improved growth rate, feed intake and efficiency, and livability (Campbell et al., 2006).

Substantially more research has been conducted to assess the effects of SDAP inclusion in swine diets, especially during the postweaning period. Spray-dried animal plasma has been routinely added to the diets of weanling piglets to improve performance, feed efficiency, and overall health (Campbell et al., 2019). A 6% inclusion level of SDAP in piglet diets during the first 2 weeks postweaning has been suggested as optimal, with a positive impact on weight gain and feed intake (van Dijk et al., 2001). Possible modes of action include increased diet palatability associated with SDAP (Ermer et al., 1994; van Dijk et al., 2001), but there is also evidence to suggest a positive influence of SDAP on gastrointestinal health, through Ig factors or the presence of antibodies that can inhibit or decrease pathogen colonization in the gastrointestinal tract (Owusu-Asiedu et al., 2002; Zhao et al., 2008). Data from swine thus support the potential for benefits associated with inclusion of SDAP in poultry diets.

It has been shown that apparent digestibility and standardized ileal AA digestibility (**SIAAD**) of diets and individual feed ingredients is lower in chicks at young ages (0–10 d) and increases with age, reaching a plateau at approximately 14 to 15 d of age (Batal and Parsons, 2002; Adedokun et al., 2008). Thus, an ingredient which is expected to be highly digestible, such as SDAP, may be particularly beneficial in diets of very young (0–10 d of age) broiler chicks. With the potential for SDAP to improve early growth performance and positively affect the gastrointestinal tract, especially in birds fed diets without growth-promoting antibiotics and raised under challenging environmental conditions, more research needs to be conducted to evaluate the nutritive value of SDAP for poultry, particularly regarding ME and AA digestibility values. The objective of this study was to determine the ME and AA digestibility of SDAP using 2 precision-fed rooster assays and 2 broiler chick assays with birds of different ages. Soybean meal was also evaluated to provide a reference for comparison, because it is the most common high-protein ingredient used in poultry diets.

## MATERIALS AND METHODS

The protocol for this study was reviewed and approved by the Institutional Animal Care and Use Committee (animal use protocol #19090 and 20131).

### Ingredients and Analyses

Spray-dried animal plasma was obtained from APC, Inc. (Ankeny, IA) and dehulled solvent-extracted SBM was obtained from a commercial plant in the Midwest. Analyses were conducted to determine nitrogen for CP via combustion (Method 990.03; AOAC International, 2007), crude fat (Method 920.93 A; AOAC International, 2007), acid detergent fiber (Method 973.18; AOAC International, 2007), neutral detergent fiber (Method 2002.04; AOAC International, 2007), total phosphorus by inductively coupled plasma optical emission spectroscopy (Method 985.01 A, B, and D; AOAC International, 2007), and ash (Method 942.05; AOAC International, 2007). The acid detergent fiber and neutral detergent fiber analyses included residual ash and NDF was determined following stable amylase pretreatment. Gross energy was analyzed using a bomb calorimeter (Model 6300; Parr Instruments, Moline, IL) and AA concentrations were also analyzed (Method 982.30 E [a, b, and c]; AOAC International, 2007). Except for gross energy, the above-mentioned analyses were conducted at the Agricultural Experiment Station Chemical Laboratory (University of Missouri, Columbia, MO).

### Diets and Design

Experiment 1 was conducted to determine TME_n_ of SDAP and SBM using conventional Single-Comb White Leghorn roosters in the precision-fed rooster assay. The mean BW of the roosters was approximately 2,200 g. There were 2 treatments with 4 replicates of 1 individually caged rooster per treatment; therefore, 8 total adult roosters were used. Roosters were fasted for 26 hours then subsequently precision tube-fed 30 g of a SDAP mixture (containing 50% corn) or SBM mixture (containing 50% corn). An additional 4 roosters were precision-fed 30 g of corn. The SDAP and SBM were fed as mixtures with corn to enable the SDAP, which had a highly fine and powdery texture, to be physically tube-fed. Each individual cage had a collection tray underneath for excreta collection and roosters were given water ad libitum. Excreta were quantitatively collected for 48 h after feeding, then the excreta were freeze-dried, ground, and weighed. The excreta collected were analyzed for gross energy and nitrogen as mentioned above. The TME_n_ values of the SDAP-corn, SBM-corn, and corn diets were calculated as described by Parsons et al. (1982) and the TME_n_ values for the SDAP and SBM were calculated by the difference procedure using the method of Han et al. (1976). The calculation equations are shown below:

TME_n_ of diets (kcal/g) = [gross energy consumed – (gross energy excreted by fed birds + 8.22 × nitrogen retained by fed birds) + (gross energy excreted by fasted birds+ 8.22 × nitrogen retained by fasted birds)]/feed intake (g)

where gross energy consumed (kcal) = diet intake (g) × gross energy of the diet (kcal/g); gross energy excreted by fed or fasted birds (kcal) = excreta output (g) × gross energy of excreta (kcal/g); 8.22 = gross energy (kcal) of uric acid per g of nitrogen (Hill and Anderson, 1958); nitrogen retained by fed or fasted birds (g) = diet intake (g) × diet nitrogen (%) - excreta output (g) × excreta nitrogen (%).

The TME_n_ values of SDAP and SBM were then calculated by difference as shown below:

TME_n_ for SDAP and SBM (kcal/g) = TME_n_ of ground corn reference diet – [(TME_n_ of ground corn reference diet – TME_n_ of test SDAP or SBM diet)/proportion of SDAP or SBM substituted into the corn reference diet]

The kcal/g values were then converted to kcal/kg by multiplying by 1,000.

Experiment 2 was conducted to determine standardized AA digestibility of SDAP and SBM using the precision-fed rooster assay. The number of birds and procedures were the same as Experiment 1 except cecectomized roosters were used. Collected excreta were analyzed for AA as described above. Basal endogenous AA concentrations were determined using roosters that were fasted for 48 h and then standardized AA digestibility values were calculated by the method of Engster et al. (1985) using the equations below:

Standardized AA digestibility of diets (%) =  [(AA consumed - AA excreted by fed birds +  AA excreted by fasted birds)/AA consumed] × 100 where AA consumed (g) = diet intake (g) × AA in diet (%); AA excreted by fed birds (g) =  excreta output (g) × AA in excreta (%); AA excreted by fasted birds =  excreta output (g) × AA in excreta (%).

The standardized AA digestibility values for the SDAP and SBM specifically were then calculated by difference using the equation:

Standardized AA digestibility of SDAP or SBM (%) = standardized AA digestibility of the ground corn reference diet –[(standardized AA digestibility of the ground corn reference diet - standardized AA digestibility of SDAP or SBM mixture diet with corn)/proportion of SDAP or SBM AA substituted into the mixture diet with corn].

Experiments 3 and 4 were conducted using Ross 708 male broiler chicks (Aviagen Group; Huntsville, AL). For both experiments, chicks were housed in Petersime starter batteries with raised wire floors in a temperature-controlled room and had ad libitum access to water and feed, which was provided in mash form. Experiment 3 was conducted to determine AME_n_ of SDAP and SBM. Chicks were fed a standard corn-SBM-based pretest diet from 0 to 6 d of age (Table 1). A 3 × 2 factorial arrangement of treatments (3 diets, 2 ages) was used to determine AME_n_. On d 7 of age, 126 chicks with a mean initial BW of 146 g were allotted to 6 replicate pens of 7 chicks per pen and fed 1 of 3 experimental diets, which consisted of a corn-SBM-based reference diet, and 2 diets in which the respective test ingredient (SDAP or SBM) was added at the expense of 30% of the complete reference diet (Table 1). Titanium dioxide was added to all diets as an indigestible marker. Chicks and feed were weighed for determination of weight gain and feed intake. Excreta were collected on d 9 and 10. Approximately 10 g of excreta were collected on trays covered with clean wax paper under the cages. On d 11, chicks were switched back to the corn-SBM-based pretest diet and the number of chicks per pen was randomly reduced from 7 to 5 to provide more space per bird for the remainder of the experiment. On d 18 of age, 90 chicks (6 replicate pens of 5 birds per pen) with a mean initial BW of 732 g were again fed 1 of the 3 experimental diets, with each pen receiving the same experimental diet as the earlier period (7–10 d of age). Chicks and feed were weighed for determination of weight gain and feed intake. Excreta were collected on d 20 and 21 and were freeze-dried, weighed, ground, and analyzed. The diets and freeze-dried excreta were analyzed for gross energy and nitrogen as described earlier and for titanium (Myers et al., 2004). The AME_n_ of each diet was then calculated at both 10 and 21 d of age using the method of Hill and Anderson (1958) and the AME_n_ of the SDAP and SBM were calculated by the difference procedure, using the method of Han et al. (1976).

$$\text{AM}E_{n}\text{of}\text{diets}\left(\text{kcal}/g\right)=\text{Ediet}-\text{Eexcreta}-8.22\times \text{nitrogen}\text{retained}$$

where Ediet = gross energy of diet (kcal/g); Eexcreta = gross energy in excreta (kcal/g) × [titanium in diet (%) / titanium in excreta (%)]; nitrogen retained (per g of diet) = nitrogen (per gram of diet) – [nitrogen (per g of excreta) × titanium in diet (%)/titanium in excreta (%)].

**Table 1:** Ingredient composition of pretest diet in Experiments 3 and 4, and corn-soybean meal-based reference diet in Experiment 3 (%; as-fed basis).

Ingredient	Pretest diet	Reference diet
Corn	52.85	58.39
Soybean meal	37.50	35.35
Pork meat and bone meal	2.00	
Soybean oil	4.00	2.04
Limestone	1.10	1.12
Dicalcium phosphate	1.50	1.24
Salt	0.40	0.45
L-Lys HCl		0.17
DL-Met	0.20	0.30
L-Thr		0.08
Vitamin mix^1^	0.20	0.20
Mineral mix^2^	0.15	0.15
Choline chloride (60%)	0.10	0.10
Phytase^3^		0.01
TiO_2_		0.40
Analyzed values		
DM		87.6
Crude protein		18.3
Fat		2.87
Ash		5.65
Neutral detergent fiber		7.23
Calcium		0.95
Total phosphorus		0.56
Sodium		0.18

1 Provided per kilogram of diet: retinyl acetate, 4,400 IU; cholecalciferol, 25µg; DL-α-tocopheryl acetate, 11 IU; vitamin B_12_, 0.01 mg; riboflavin, 4.41 mg; D-pantothenic acid, 10 mg; niacin, 22 mg; menadione sodium bisulfate, 2.33 mg.

2 Provided as milligrams per kilogram of diet: manganese, 75 from MnSO_4_·H_2_O; iron, 75 from FeSO_4_·H_2_O; zinc, 75 from ZnO; copper, 5 from CuSO_4_·5H_2_O; iodine, 75 from ethylene diamine dihydroiodide; selenium, 0.1 from Na_2_SeO_3_.

3 Optiphos 2000 (Huvepharma; Sofia, Bulgaria). Supplied 300 FTU/kg of phytase.

The AME_n_ values for the SDAP and SBM specifically were then calculated by difference using the equation:

$$\text{AM}E_{n}\text{for}\text{SDAP}\text{and}\text{SBM}\left(\text{kcal}/g\right)=\text{AM}E_{n}\text{of}\text{corn}-\text{SBM}\text{reference}\text{diet}-\left[\left(\text{AM}E_{n}\text{of}\text{corn}-\text{SBM}\text{reference}\text{diet}-\text{AM}E_{n}\text{of}\text{test}\text{SDAP}\text{or}\text{SBM}\text{diet}\right)/\text{proportion}\text{of}\text{SDAP}\text{or}\text{SBM}\text{substituted}\text{into}\text{the}\text{corn}-\text{SBM}\text{reference}\text{diet}\right]$$

Experiment 4 was conducted to determine apparent ileal AA digestibility (**AIAAD**) and SIAAD of SDAP and SBM. Chicks were fed a standard corn-SBM-based pretest diet from 0 to 6 d of age (Table 1). A 2 × 2 factorial arrangement of treatments (2 diets, 2 ages) was used. For the first experimental period, on d 7 of age, 168 chicks with a mean initial BW of 112 g were allotted to 12 replicate pens of 7 chicks per pen and fed 1 of 2 experimental diets consisting of a cornstarch-dextrose-SDAP diet and a cornstarch-dextrose-SBM diet from 7 to 10 d of age (Table 2). Both diets were formulated to contain 20% dietary protein, with SDAP or test SBM as the only source of protein, and titanium dioxide was added as an indigestible marker. On d 10, chicks were euthanized using CO_2_ gas and the digesta contents from the ileum (Meckel's diverticulum to the ileal-cecal junction) were collected using a combination of flushing with water and gentle squeezing, and freeze-dried. Ileal digesta from 2 replicate pens were pooled together to provide enough sample for analysis, yielding 6 replicate pen values per treatment for statistical analysis.

**Table 2:** Ingredient composition of diets in Experiment 4 (%; as-fed basis).

	Dietary treatments	
Ingredient	SDAP^1^	SBM^2^
Cornstarch	30.32	24.66
Dextrose	30.31	24.65
SDAP^1^	25.00	0.00
SBM^2^	0.00	41.24
Soybean oil	5.00	5.00
Cellulose	5.00	0.00
Limestone	1.87	0.95
Dicalcium phosphate	0.70	1.95
Salt	0.00	0.40
K_2_CO_3_	0.45	0.00
MgO	0.20	0.00
Vitamin mix^3^	0.20	0.20
Mineral mix^4^	0.15	0.15
Choline chloride (60%)	0.30	0.30
TiO_2_	0.50	0.50
Analyzed values		
DM	92.7	91.9
Crude protein	19.6	20.9
Fat	3.25	3.94
Ash	5.23	6.08
Neutral detergent fiber	2.84	4.29
Calcium	1.06	0.97
Total phosphorus	0.51	0.67
Sodium	0.70	0.17

1 Spray-dried animal plasma (APC, Inc.; Ankeny, IA).

2 Soybean meal.

3 Provided per kilogram of diet: retinyl acetate, 4,400 IU; cholecalciferol, 25µg; DL-α-tocopheryl acetate, 11 IU; vitamin B_12_, 0.01 mg; riboflavin, 4.41 mg; D-pantothenic acid, 10 mg; niacin, 22 mg; menadione sodium bisulfate, 2.33 mg.

4 Provided as milligrams per kilogram of diet: manganese, 75 from MnSO_4_·H_2_O; iron, 75 from FeSO_4_·H_2_O; zinc, 75 from ZnO; copper, 5 from CuSO_4_·5H_2_O; iodine, 75 from ethylene diamine dihydroiodide; selenium, 0.1 from Na_2_SeO_3_.

For the second experimental period (18–21 d of age), chicks were fed a standard corn-SBM-based pretest diet from 0 to 17 d of age (Table 1). On d 18 of age, 60 chicks with a mean initial BW of 512 g were allotted to 6 replicate pens of 5 chicks per pen and fed 1 of the same 2 experimental diets (Table 2). On d 21, chicks were euthanized using CO_2_ gas and the digesta contents from the ileum (Meckel's diverticulum to the ileal-cecal junction) were collected using a combination of flushing with water and gentle squeezing, and freeze-dried. Diets and freeze-dried ileal digesta collected on 10 and 21 d of age were analyzed for AA and titanium. The AIAAD and SIAAD values were calculated as shown below:

AIAAD(%)=[(AAdiet−AAilealdigesta)/AAdiet]×100

where AAdiet = AA in the diet (%); AA ileal digesta = AA in ileal digesta (%) × titanium in diet (%)/titanium in ileal digesta (%).

The AIAAD values were then standardized using the basal ileal endogenous AA flow values (IEAA; mg/kg DM intake) for 21 d old broiler chickens fed a nitrogen-free diet from the study of Adedokun et al. (2007) and then the SIAAD values were calculated as described by Adedokun et al. (2009):

SIAAD(%)=AIAAD(%)+[100×basalIEAAflow(g/kgDMintake)/AAindiet(g/kgDM)]

### Statistical Analysis

Data from all assays were subjected to ANOVA (SAS Institute; Cary, NC) for a completely randomized design. For Experiments 1 and 2, the statistical significance of differences between individual treatments was assessed using the *P* value for the model in the ANOVA since there were only 2 treatments. The experimental unit was the individual rooster. For Experiment 3, growth performance data and AME_n_ values for diets were analyzed as a 3 × 2 factorial arrangement of treatments with diet (reference diet, 30% SDAP, and 30% SBM) and age (d 7–10 and d 18–21) as main effect variables. For AME_n_ of ingredients in Experiment 3 and SIAAD in Experiment 4, data were analyzed as a 2 × 2 factorial arrangement of treatments with ingredient (SDAP and SBM) and age (d 7–10 and d 18–21) as main effect variables. In Experiments 3 and 4, the pen served as the experimental unit. Also, for Experiments 3 and 4, pairwise treatment comparisons were conducted using the least significant difference test (Carmer and Walker, 1985) when the interaction between main effects was significant. The probability level for significant differences for all comparisons was considered at *P* < 0.05.

## RESULTS AND DISCUSSION

### Nutrient Composition

Table 3 contains the analyzed nutrient composition of SDAP and SBM. Crude protein values for SDAP and SBM were similar to the values reported in NRC (2012; 1994) at 84.6% and 53.7%, respectively. As expected, SBM contained a greater level of fiber than SDAP. The ingredients were also analyzed for P, with SDAP and SBM containing levels similar to what is reported in the NRC (1994; 2012). Crude fat in SDAP was much lower than a previously reported value for dried bovine plasma of 1.5% fat (Howell and Lawrie, 1983). Compared with values reported by King et al. (2005), SDAP used in the current study was greater in DM, CP, and P, but similarly very low in crude fat. The reason for the difference in composition for some nutrients and components for SDAP among studies is unknown.

**Table 3:** Analyzed composition and TME_n_ of spray-dried animal plasma and soybean meal in Experiment 1 (DM basis^1^).

	Spray-dried animal plasma^2^	Soybean meal^2^	SEM
Crude protein (%)	84.7	52.4	
Crude fat (%)	0.02	0.54	
Acid detergent fiber (%)	0.7	9.8	
Neutral detergent fiber (%)	3.1	10.7	
P (%)	1.41	0.67	
Ash (%)	8.62	6.92	
Gross energy (kcal/kg)	5,192	4,741	
TME_n_ (kcal/kg)^2^^,^^3^	3,743	2,669	114

1 DM values for spray-dried animal plasma and soybean meal were 92.9% and 90.0%, respectively.

2 Spray-dried animal plasma and soybean meal were fed to conventional roosters as a 50% blend with corn; TME_n_ values were calculated by the difference procedure, factoring out the corn contribution.

3 Values are means of 4 individually-caged conventional roosters. The probability value for the model from the ANOVA was *P* < 0.0001 indicating that the TME_n_ of spray-dried animal plasma was significantly higher than soybean meal.

### Experiment 1

Gross energy was numerically greater for SDAP compared with SBM (Table 3). In a study using swine, Almeida et al. (2013) evaluated SDAP and obtained a gross energy of 5,173 kcal/kg DM, which is similar to SDAP in the current study. The TME_n_ of SDAP was greater (*P* < 0.05) than SBM, and the greater TME_n_ value obtained in Experiment 1 for SDAP than SBM using the precision-fed rooster assay is similar to the results by Norberg et al. (2004), who obtained TME_n_ values for plasma protein and SBM in ducks of 3,555 and 2,930 kcal/kg DM, respectively. The higher TME_n_ content of SDAP compared with SBM is probably due mainly to the much higher digestible protein content and lower fiber content of SDAP (Table 3).

### Experiment 2

Table 4 contains standardized AA digestibility values and digestible AA concentrations for SDAP and SBM determined using the precision-fed rooster assay. Standardized digestibility values for all AA in SDAP were greater (*P* < 0.05) than SBM. Likewise, mean AA digestibility for SDAP was greater than SBM (94% and 86%, respectively). The largest differences for standardized AA digestibility between SDAP and SBM were for Cys, Ala, Thr, and Val, all of which were at least 10 percentage units greater for SDAP. Due to its greater total AA content and standardized AA digestibility values, SDAP was calculated to contain greater concentrations of digestible AA than SBM.

**Table 4:** Total amino acids, standardized amino acid digestibility values, and digestible amino acid concentrations for spray-dried animal plasma and soybean meal in Experiment 2 (%, DM basis).

	Spray-dried animal plasma^1^			Soybean meal^1^			
Amino acid	Total	Digest. value^2^	Digest. conc.^3^	Total	Digest. value^2^	Digest. conc.^3^	SEM^4^
Met	1.03	91	0.94	0.80	83	0.67	1.4
Cys	2.94	94	2.75	0.79	76	0.60	1.6
Lys	7.61	93	7.07	3.32	85	2.81	1.1
Thr	5.54	95	5.25	2.07	83	1.72	0.9
Val	5.97	95	5.68	2.54	85	2.16	1.2
Arg	4.95	95	4.70	3.66	90	3.30	0.8
Ile	2.71	91	2.48	2.61	86	2.25	1.0
Asp	8.60	94	8.06	5.97	87	5.17	0.6
Ser	5.21	94	4.89	2.26	85	1.91	0.8
Glu	11.91	95	11.26	9.39	90	8.46	0.6
Pro	4.52	95	4.28	2.52	87	2.20	1.0
Ala	4.11	93	3.83	2.29	81	1.86	1.4
Leu	8.01	95	7.60	4.01	86	3.43	1.1
Tyr	4.26	95	4.05	1.72	86	1.47	1.0
Phe	4.49	95	4.25	2.66	87	2.32	1.0
His	2.59	93	2.42	1.37	87	1.19	0.8
Trp	1.70	97	1.64	0.77	92	0.71	0.4
Mean		94			86		

1 Spray-dried animal plasma and soybean meal were fed to cecectomized roosters as a 50% blend with corn; amino acid digestibility values were calculated by the difference procedure, factoring out the corn contribution.

2 Standardized digestibility values are means of 4 individually caged cecectomized roosters.

3 Digestible concentrations = (total × standardized digestibility value)/100.

4 SEM for standardized digestibility values. The probability value for the model from the ANOVA was *P* < 0.0001 for all amino acids, indicating that standardized digestibility values for all amino acids in spray–dried animal plasma were significantly higher than soybean meal.

Standardized AA digestibility values of SDAP in this experiment were somewhat greater than SIAAD values listed for blood plasma for pigs (NRC, 2012), including Met (84%), Cys (85%), Lys (87%), Thr (80%), Ile (85%), Val (82%), Trp (92%), and Arg (91%). These differences may be due to differences in species or ingredient batch and/or processing method. Standardized AA digestibility values determined herein for SBM were generally lower than values for poultry in the NRC (1994). The reason for the somewhat lower values in the current study is unknown.

Almeida et al. (2013) evaluated the SIAAD of several blood products (spray-dried animal blood, spray-dried blood cells, and spray-dried plasma protein) in pigs. When fed to weanling pigs, the mean SIAAD for total AA was found to be high for the spray-dried animal blood, spray-dried blood cells, and spray-dried plasma protein (100%, 95%, and 98%, respectively). These high SIAAD values with pigs are in agreement with high standardized AA digestibility values for SDAP in cecectomized roosters from Experiment 2.

### Experiment 3

Weight gain and feed intake were affected (*P* < 0.05) by diet and age, and there were significant interactions for both. However, no differences (*P* > 0.05) between birds fed the SDAP and SBM diets were noted, with the exception of feed intake from d 18 to 21 of age, with birds fed the SBM diet consuming more feed. The AME_n_ on d 10 and 21 were greater (*P* < 0.05) for the diet containing 30% SDAP than the diet containing 30% test SBM and the reference diet (Table 5). Similarly, for the ingredients, AME_n_ for SDAP was greater (*P* < 0.05) than SBM on d 10 and d 21. In addition, AME_n_ for both ingredients increased (*P* < 0.05) with age. However, although the increase in AME_n_ for SBM between 10 and 21 d was numerically greater than that of SDAP, the interaction of ingredient and age was not significant (*P* > 0.05).

**Table 5:** Weight gain, feed intake, and AME_n_ values for spray-dried animal plasma and soybean meal in Experiment 3.

Response	Age	Reference diet	30% SDAP^1^	30% Test SBM^1^		Probability, *P*		
					SEM	Diet	Age	Diet × Age
Weight gain, g/chick^2^	d 7 to 10	157.8^b^	155.9^bc^	134.5^c^	7.5	0.0022	<0.0001	0.0003
	d 18 to 21	163.9^b^	222.0^a^	202.2^a^				
Feed intake, g/chick^2^	d 7 to 10	204.5^c^	148.3^d^	167.0^d^	8.8	0.0007	<0.0001	0.0254
	d 18 to 21	292.5^ab^	273.3^b^	303.8^a^				
AME_n_ of diets, kcal/kg as-fed^2^	d 7 to 10	2,756^c^	3,003^b^	2,493^d^	28	<0.0001	<0.0001	0.0068
	d 18 to 21	2,793^c^	3,136^a^	2,724^c^				
AME_n_ of ingred. kcal/kg DM^3^	d 7 to 10	–	3,851	2,089	109	<0.0001	<0.0001	0.0997
	d 18 to 21	–	4,239	2,849				

^a-d^Values within the same response criteria (across column and row) with no common superscript differ (*P* < 0.05).

1 Response values for d 7 to 10 of age for spray-dried animal plasma (SDAP) and soybean meal (SBM) are means of 6 replicates of 7 chicks per pen. Response values for d 18 to 21 of age for spray-dried animal plasma (SDAP) and soybean meal (SBM) are means of 6 replicates of 5 chicks per pen.

2 Analyzed as a 3 × 2 factorial arrangement of treatments, with 3 diets and 2 ages.

3 Analyzed as a 2 × 2 factorial arrangement of treatments, with 2 ingredients and 2 ages.

As mentioned earlier, the AME_n_ on d 10 and 21 for SBM were 2,089 and 2,849 kcal/kg DM, respectively; the former value is considerably lower, and the latter value is slightly greater, than the value of 2,711 kcal/kg DM reported in the NRC (1994). The reason for the d 10 value being lower than the NRC (1994) value is probably due to the reduced ability to digest nutrients and lower AME_n_ values for a corn-SBM diet at young ages as previously reported by Noy and Sklan (1995) and Batal and Parsons (2002).

A factor that could have possibly affected AME_n_ values is that the chicks used for the determination of AME_n_ at 21 d had been fed the SDAP and SBM test diets earlier from 7 to 10 d of age. Thus, it is possible that there could have been some carryover effect of the diets fed from 7 to 10 d on the AME_n_ values determined at 21 d. The older birds had been fed a corn-SBM diet from 10 to 17 d an attempt to minimize any such possible carryover effects. This 7 d feeding period for the corn-SBM diet exceeded the 2-d feeding period used previously in Latin Square design experiments to determine digestibility of AA in different diets for laying hens (Zuber and Rodehutscord, 2017; Zuber et al., 2017). In addition, there were no significant differences in BW gain for either the 7 to 10 d or 18 to 21 d feeding periods between chicks fed the 30% SDAP or 30% SBM diets. Thus, these collective observations support that any carryover effects of feeding the SDAP or SBM diets from 7 to 10 d, if occurring, were not large.

The rooster assay (Experiment 1) may have underestimated the energy difference between SDAP and SBM for chicks, and particularly young chicks. There was a 1,074 kcal/kg DM difference in TME_n_ for the SDAP and SBM in roosters in Experiment 1. Numerically greater and significant differences in AME_n_ between SDAP and SBM were observed in chicks (Experiment 3), with the difference being 1,762 and 1,390 kcal/kg DM at d 10 and 21, respectively. These observations are not unexpected because previous studies have shown that energy digestibility or AME_n_ increases with age during the first 2 to 3 weeks after hatching (Noy and Sklan, 1995; Batal and Parsons, 2002). Thus, AME_n_ of the less digestible SBM would be expected to be substantially greater in the adult roosters than the chicks, particularly at the youngest age of 10 d. The latter difference would be expected to be smaller for the more highly digestible SDAP. The results of this experiment indicate that SDAP is a highly digestible energy source for broiler chicks even at a young age and, thus, may be a particularly good ingredient in diets of very young chicks. Interestingly, even though SDAP had a high AME_n_ at 10 d, the AME_n_ of SDAP increased with age between 10 and 21 d.

### Experiment 4

All AIAAD (Table 6) and SIAAD (Table 7) values for SDAP were greater (*P* < 0.05) than values for SBM at d 10, with SDAP having a mean AIAAD of 94% compared with 82% for SBM. Similar results were observed for d 21, with SDAP and SBM having mean AIAAD values of 95% and 85%, respectively. Thus, as observed in Experiment 2 with roosters, large differences for AA digestibility between SDAP and SBM were observed. The AIAAD values compared with SIAAD values for SDAP and the AIAAD values compared with SIAAD values for SBM at the same bird age were similar, with mean AA digestibility values differing by only 2 percentage units.

**Table 6:** Apparent ileal amino acid digestibility values and digestible amino acid concentrations for spray-dried animal plasma and soybean meal determined at d 10 and d 21 of age in Experiment 4 (%, DM basis).

Amino acid	d 10 of age^1^				d 21 of age^2^				SEM^4^	Digestibility value probability, *P*^4^		
	SDAP		SBM		SDAP		SBM					
	Digest. value	Digest. conc.^3^	Digest. value	Digest. conc.^3^	Digest. value	Digest. conc.^3^	Digest. value	Digest. conc.^3^		Ingred.	Age	Ingred. × age
Met	92	0.94	84	0.67	95	0.98	89	0.71	1.1	<0.0001	0.0003	0.4214
Cys	95^a^	2.78	63^c^	0.50	94^a^	2.76	70^b^	0.55	0.9	<0.0001	0.0026	0.0003
Lys	95	7.22	82	2.74	96	7.34	87	2.90	0.9	<0.0001	0.0013	0.0654
Thr	92^a^	5.12	74^c^	1.54	93^a^	5.15	79^b^	1.64	0.7	<0.0001	0.0013	0.0074
Val	94^a^	5.59	80^c^	2.03	94^a^	5.61	83^b^	2.11	0.7	<0.0001	0.0100	0.0338
Arg	95	4.68	89	3.27	97	4.79	92	3.35	0.7	<0.0001	0.0025	0.9676
Ile	91	2.47	82	2.15	94	2.54	86	2.23	0.8	<0.0001	0.0025	0.7452
Asp	93	8.03	81	4.80	94	8.05	83	4.94	0.6	<0.0001	0.0524	0.1048
Ser	94^a^	4.88	79^c^	1.79	93^a^	4.85	82^b^	1.86	0.7	<0.0001	0.0475	0.0114
Glu	94	11.19	88	8.21	96	11.37	89	8.40	0.6	<0.0001	0.0072	0.7110
Pro	93	4.21	83	2.10	94	4.23	85	2.15	0.5	<0.0001	0.0297	0.1189
Ala	94	3.85	81	1.85	95	3.92	85	1.94	0.8	<0.0001	0.0010	0.1409
Leu	95	7.61	83	3.32	96	7.70	86	3.43	0.7	<0.0001	0.0060	0.2290
Tyr	95^a^	4.05	83^c^	1.43	96^a^	4.08	86^b^	1.48	0.6	<0.0001	0.0026	0.0433
Phe	94	4.24	84	2.24	95	4.28	87	2.31	0.6	<0.0001	0.0104	0.2741
His	95	2.45	85	1.16	96	2.48	87	1.20	0.6	<0.0001	0.0048	0.1690
Trp	96^a^	1.63	85^c^	0.65	96^a^	1.64	89^b^	0.68	0.6	<0.0001	0.0012	0.0082
Mean	94		82		95		85					

^a-c^Digestibility values within the same row with no common superscripts differ (*P* < 0.05).

1 Apparent digestibility values at d 10 of age for spray-dried animal plasma (SDAP) and soybean meal (SBM) are means of 6 replicates; each replicate consists of a pooled sample from 2 pens of 7 chicks per pen.

2 Apparent digestibility values at d 21 of age are means of 6 replicate pens of 5 chicks per pen.

3 Digestible concentrations = (total (Table 4) AA concentration × apparent digestibility value)/100.

4 SEM and probability values for apparent digestibility values; analyzed as a 2 × 2 factorial arrangement of treatments, with 2 ingredients and 2 ages.

**Table 7:** Standardized ileal amino acid digestibility values and digestible amino acid concentrations for spray-dried animal plasma and soybean meal determined at d 10 and d 21 of age in Experiment 4 (%, DM basis).

Amino acid	d 10 of age^1^				d 21 of age^2^				SEM^4^	Digestibility value probability, *P*^4^		
	SDAP		SBM		SDAP		SBM					
	Digest. value	Digest. conc.^3^	Digest. value	Digest. conc.^3^	Digest. value	Digest. conc.^3^	Digest. value	Digest. conc.^3^		Ingred.	Age	Ingred. × age
Met	94	0.97	86	0.68	97	1.01	91	0.73	1.1	<0.0001	0.0003	0.4209
Cys	97^a^	2.84	67^c^	0.53	96^a^	2.82	74^b^	0.58	0.9	<0.0001	0.0026	0.0003
Lys	96	7.30	84	2.78	97	7.41	89	2.94	0.9	<0.0001	0.0013	0.0654
Thr	95^a^	5.24	78^c^	1.61	95^a^	5.27	83^b^	1.70	0.7	<0.0001	0.0014	0.0075
Val	95^a^	5.69	82^c^	2.09	96^a^	5.71	86^b^	2.18	0.7	<0.0001	0.0100	0.0338
Arg	96	4.76	91	3.31	98	4.87	93	3.39	0.7	<0.0001	0.0025	0.9655
Ile	94	2.54	84	2.20	96	2.61	87	2.28	0.8	<0.0001	0.0024	0.7459
Asp	95	8.18	82	4.89	95	8.19	84	5.02	0.6	<0.0001	0.0527	0.1049
Ser	96^a^	4.99	82^c^	1.85	95^a^	4.97	85^b^	1.92	0.7	<0.0001	0.0476	0.0114
Glu	96	11.37	89	8.32	97	11.55	91	8.51	0.6	<0.0001	0.0071	0.7100
Pro	95	4.31	86	2.16	96	4.33	87	2.20	0.5	<0.0001	0.0295	0.1194
Ala	96	3.93	83	1.90	97	4.00	87	1.99	0.8	<0.0001	0.0010	0.1406
Leu	96	7.71	84	3.38	98	7.81	87	3.50	0.7	<0.0001	0.0060	0.2294
Tyr	97^a^	4.11	85^c^	1.46	97^a^	4.14	88^b^	1.51	0.6	<0.0001	0.0026	0.0434
Phe	96	4.30	86	2.28	97	4.35	88	2.34	0.6	<0.0001	0.0104	0.2752
His	96	2.49	86	1.17	97	2.51	89	1.21	0.6	<0.0001	0.0047	0.1692
Trp	97^a^	1.64	86^c^	0.66	97^a^	1.65	90^b^	0.69	0.6	<0.0001	0.0012	0.0081
Mean	96		84		97		87					

^a-c^Digestibility values within the same row with no common superscripts differ (*P* < 0.05).

1 Standardized digestibility values at d 10 of age for spray-dried animal plasma (SDAP) and soybean meal (SBM) are means of 6 replicates; each replicate consists of a pooled sample from 2 pens of 7 chicks per pen.

2 Standardized digestibility values at d 21 of age are means of 6 replicate pens of 5 chicks per pen.

3 Digestible concentrations = (total (Table 4) AA concentration × standardized digestibility value)/100.

4 SEM and probability values for standardized digestibility values; analyzed as a 2 × 2 factorial arrangement of treatments, with 2 ingredients and 2 ages.

The AIAAD and SIAAD values at d 10 were lower (*P* < 0.05) than values at d 21 for SBM, but only small differences were generally observed between ages for SDAP (Tables 6 and 7, respectively). There were significant main effects (*P* < 0.05) of ingredient and age for all AA. In addition, significant interactions (*P* < 0.05) between age and ingredient were observed for Cys, Thr, Val, Ser, Tyr, and Trp. Why there was a significant interaction for some AA and not others is unknown. The interaction occurred because the age-related increase in AIAAD and SIAAD was larger for SBM than SDAP. Adedokun et al. (2008) found that the effect of age on SIAAD values varied among ingredients, with values being increased with age for corn and DDGS but not for SBM and canola meal. The results of this experiment indicate that SDAP has higher AIAAD and SIAAD (*P* < 0.05) compared with SBM at both d 10 and d 21, and these results agree with Experiment 2 in which standardized AA digestibility of SDAP was higher than SBM (*P* < 0.05) in older precision-fed roosters.

The SIAAD values for SDAP and SBM were calculated because SIAAD values are generally considered to be superior to AIAAD values (Lemme et al., 2004; Adedokun et al., 2011). As mentioned earlier, the SIAAD values were calculated using ileal endogenous AA values from broiler chickens fed a nitrogen-free diet in an earlier study by Adedokun et al. (2007). This procedure of using published endogenous values to calculate SIAAD has been used previously (Adedokun et al., 2009; Kim et al., 2011; Kim et al., 2012) and has also been used for calcium digestibility (David et al., 2021). Using previously published values reduces the number of animals needed in experiments and particularly decreases the number of animals that need to be fed a severely nutrient deficient nitrogen-free diet, which facilitates and is sometimes necessary to obtain approval by the Institutional Animal Care and Use Committee. However, this procedure of using previously published values could introduce some error into the SIAAD values since it is well-known that several factors can influence ileal endogenous AA losses (Adedokun et al., 2011). Hopefully, any such error was not large. The latter is supported by the observations that the AIAAD and SIAAD values for SDAP and SBM differed by only a small amount, 2 percentage units or less, indicating that the effect of the ileal endogenous AA correction was small. In addition, the magnitude of the differences between AA digestibility values for SDAP and SBM were identical for both AIAAD and SIAAD. The observation that AIAAD and SIAAD values in the current study were similar is in agreement with previous research that has shown that apparent and standardized values for high protein ingredients generally do not differ by a large amount, whereas the difference between apparent and standardized values is much larger for a low protein ingredient such corn (Adedokun et al., 2009; Kim et al., 2012). Both the SDAP and SBM evaluated in the current study were high protein ingredients.

The high AME_n_ and AA digestibility for SDAP in young chicks (7–10 d) is particularly interesting and noteworthy. Several studies have shown that energy and AA digestibility are reduced in young chicks during the first 15 d of life (Noy and Sklan, 1995; Batal and Parsons, 2002; Adedokun et al., 2008). Noy and Sklan (1995) reported that after 10 d posthatch, passage rate of feed through the intestines decreased by approximately 33%. Pancreatic enzyme concentrations increased rapidly with age; thus, proteolysis may not be sufficient in the early posthatch period to optimally hydrolyze exogenous and endogenous proteins in the small intestine in young chicks (Noy and Sklan, 1995). Thus, including an ingredient such as SDAP, which is highly digestible even at very young ages (7–10 d), in the diets of young chicks should be beneficial. Beski et al. (2016) reported that a low inclusion level (1%) of spray-dried porcine plasma in chick diets during the first 10 d posthatch had beneficial performance and production effects, and that the increase in performance persisted until marketing.

In summary, these experiments indicate that SDAP is a highly digestible protein source for poultry diets. Due to the high AME_n_ and high SIAAD observed even in young chicks (7–10 d), SDAP may be a particularly beneficial ingredient to include in chick starter diets.

## Disclosures

There is no conflict of interest in the publishing of metabolizable energy and amino acid digestibility in spray-dried animal plasma using broiler chick and precision-fed rooster assays.
